# Libman-Sacks endocarditis and associated cerebrovascular disease: The role of medical therapy

**DOI:** 10.1371/journal.pone.0247052

**Published:** 2021-02-16

**Authors:** Carlos A. Roldan, Wilmer L. Sibbitt, Ernest R. Greene, Clifford R. Qualls, Rex E. Jung

**Affiliations:** 1 Department of Medicine, Divisions of Cardiology and Rheumatology, University of New Mexico School of Medicine, Albuquerque, New Mexico, United States of America; 2 Department of Neurosurgery, University of New Mexico School of Medicine, Albuquerque, New Mexico, United States of America; Kaohsuing Medical University Hospital, TAIWAN

## Abstract

**Background:**

Libman-Sacks endocarditis in patients with systemic lupus erythematosus (SLE) is commonly complicated with embolic cerebrovascular disease (CVD) or valve dysfunction for which high-risk valve surgery is frequently performed. However, the role of medical therapy alone for Libman-Sacks endocarditis and associated acute CVD remains undefined.

**Objective:**

To determine in this cross-sectional and longitudinal study if conventional anti-inflammatory and anti-thrombotic therapy may be an effective therapy in SLE patients with Libman-Sacks endocarditis and associated acute CVD.

**Methods and materials:**

17 SLE patients with Libman-Sacks endocarditis detected by two-and-three-dimensional transesophageal echocardiography (TEE) and complicated with acute CVD [stroke/TIA, focal brain injury on MRI, or cognitive dysfunction] were treated with conventional anti-inflammatory and anti-thrombotic therapy for a median of 6 months and then underwent repeat TEE, transcranial Doppler, brain MRI, and neurocognitive testing for re-assessment of Libman-Sacks endocarditis and CVD.

**Results:**

Valve vegetations decreased in number, diameter, and area (all p ≤0.01); associated valve regurgitation significantly improved (p = 0.04), and valve thickening did not progress (p = 0.56). In 13 (76%) patients, valve vegetations or valve regurgitation resolved or improved in number and size or by ≥1 degree, respectively, as compared to 4 (24%) patients in whom vegetations or valve regurgitation persisted unchanged or increased in size or by ≥1 degree (p = 0.03). Also, cerebromicroembolism, lobar and global gray and white matter cerebral perfusion, ischemic brain lesion load, and neurocognitive dysfunction resolved or significantly improved (all p ≤0.04).

**Conclusion:**

These preliminary data suggest that combined conventional anti-inflammatory and antithrombotic therapy may be an effective treatment for Libman-Sacks endocarditis and its associated CVD and may obviate the need for high-risk valve surgery.

## Introduction

Libman-Sacks endocarditis is common in systemic lupus erythematosus (SLE) and is frequently complicated with 1) embolic cerebrovascular disease (CVD) manifested as cerebromicroembolism, stroke or transient ischemic attacks (TIA), ischemic brain injury on MRI, or cognitive dysfunction and disability [1–3]; 2) acute or chronic progressive valve dysfunction [4]; 3) frequent need for high risk valve surgery [5, 6]; and 4) increased morbidity and mortality [4–6]. Guidelines and experts’ consensus are available on medical therapy for most SLE-related clinical syndromes [7–9], but they do not exist for Libman-Sacks endocarditis and its associated CVD due to lack of studies on medical therapy alone and lack of comparative studies on medical versus surgical therapy. However, patients with Libman-Sacks endocarditis complicated with CVD or valve dysfunction frequently undergo valve replacement or repair with 3–5 times higher morbidity and mortality than in general populations without SLE [4–6]. Based on the immune-mediated inflammatory and thrombotic pathogenesis of Libman-Sacks endocarditis and its associated thromboembolic CVD [1–3, 10–12], combined anti-inflammatory and anti-thrombotic therapy may be a reasonable and effective initial therapy for this condition. To assess this hypothesis, we conducted this cross-sectional and longitudinal study in a well-characterized cohort of SLE patients with Libman-Sacks endocarditis and acute CVD treated with conventional anti-inflammatory and anti-thrombotic therapy and who then underwent repeat cardiac and cerebrovascular clinical and imaging re-evaluations.

## Methods and materials

### Study population

This study was part of a principal study design and protocol approved by the National Institutes of Health and Institutional Review Board (IRB) of the University of New Mexico School of Medicine that determined an association of Libman-Sacks endocarditis and embolic cerebrovascular disease (CVD) in SLE [1].

The primary study included 76 patients with SLE and 26 apparently healthy volunteers age-and-sex frequency (3 patients to 1 control ratio) matched to patients to validate blinded interpretation of tests and provide a normal reference. From December 2006 to December 2012, patients were recruited by the study coordinator from 266 patients regularly followed at the outpatient rheumatology clinics or inpatient services of the University of New Mexico Health Sciences Center. Controls were recruited from available listings of volunteers in the Office of Research of the Health Sciences Center, employees of the University of New Mexico, or patients’ relatives or acquaintances. All participants provided IRB-approved informed written consent obtained by the study research coordinator. This study was conducted according to the principles of the Declaration of Helsinki.

All 102 participants underwent a standardized protocol of clinical and laboratory evaluations, transesophageal echocardiography (TEE), transcranial Doppler, brain MRI and neurocognitive testing within 1 week of enrollment.

**Fig 1:**
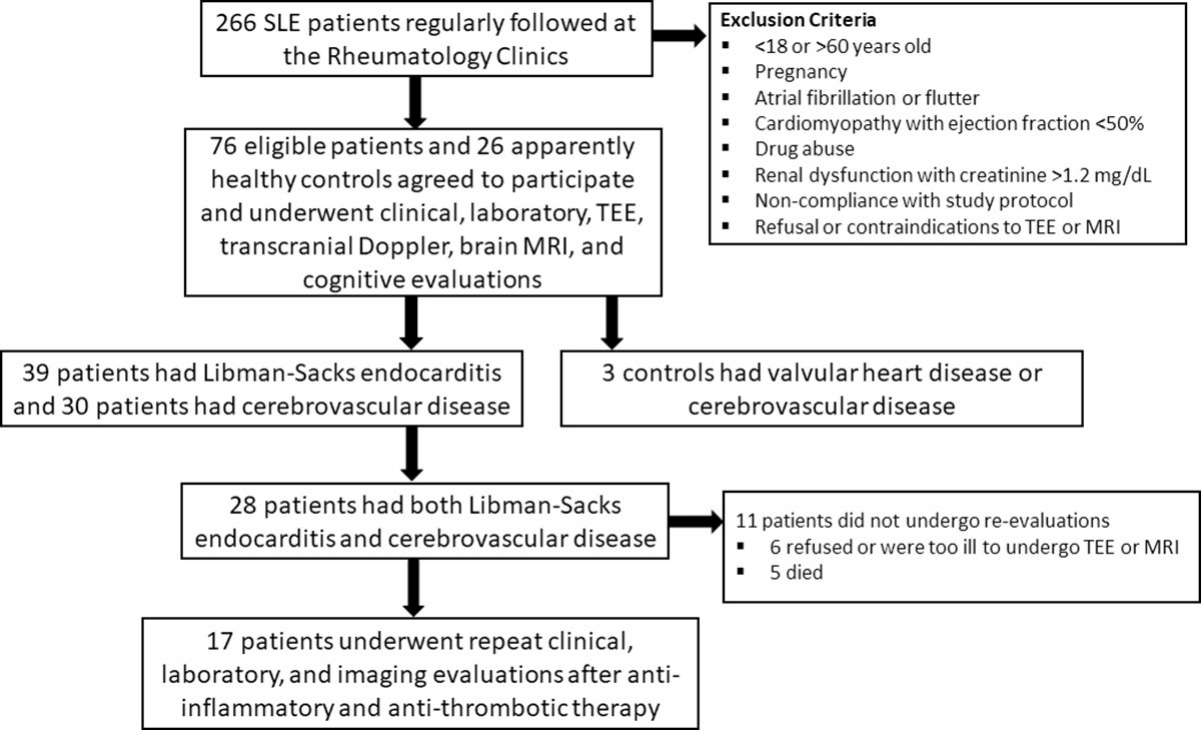
Study population recruitment, selection, evaluations, and treatment.

To assess the effect of medical therapy on Libman-Sacks endocarditis and its associated embolic CVD, 17 patients with both conditions underwent repeat clinical, laboratory, cardiac and cerebrovascular evaluations after a median of 6 months (interquartile range, 2.1–9.6) of clinically indicated and patients’ provider guided anti-inflammatory and anti-thrombotic therapy. These 17 patients [age 36 ± 12 years (range, 18–57), 14 (82%) women, 53% Hispanic, with body mass index of 27.12 ± 7.5 Kg/m^2^, age at onset of SLE 29.31 ± 12.08, and SLE duration of 7.53 ± 6.10 years] constitute this study population and their selection is summarized in **Fig 1**.

All initial and follow-up studies were coded, de-identified, randomly intermixed, and interpreted by experienced observers blinded to subjects’ clinical and imaging data, baseline findings, and each other’s readings.

Limited data on the response to medical therapy of overall vegetations’ number and area, cerebromicroembolism, and brain perfusion and lesion load was reported in the manuscript of the primary study determining an association of Libman-Sacks endocarditis with embolic CVD [1]. However, this manuscript aimed to assess the response to anti-inflammatory and anti-thrombotic therapy of Libman-Sacks endocarditis and its associated CVD expands significantly on 1) new individual data on the type of CVD and type and duration of anti-inflammatory and anti-thrombotic therapy, and 2) extensive new data on the response to medical therapy of a) parameters of inflammation and thrombogenesis, b) mitral and aortic valve vegetations’ number, area, and diameter; c) valve regurgitation and valve thickness; d) overall and mitral and aortic valve vegetations’ number, area, and diameter assessed by 3-dimensional (3D) TEE; e) cerebral perfusion and lesion load of the left and right side of each cerebral lobe and right and left cerebral hemispheres, respectively; and f) neurocognitive function on specific components of the clinical domains of memory and motor function.

### Clinical and laboratory evaluations

During initial and follow-up studies, patients were characterized with regard to general demographics, SLE activity and damage, standard autoantibodies, and parameters of inflammation, platelet activation, and fibrinolysis. SLE disease activity was determined with the Systemic Lupus Erythematosus Disease Activity Index 2000 (SLEDAI-2K) [13]. The Neuro-SLEDAI-2K is composed of the neurologic components of SLEDAI-2K (seizure, psychosis, organic brain syndrome, visual disturbance, cranial neuropathy, lupus headache, and stroke), each of which is given a score ranging from 0 to 8 and then summed. SLE damage was determined with the Systemic Lupus International Collaborating Clinics/American College of Rheumatology Damage Index (SDI) [13]. The Neuro-SDI measures the neurologic components of SDI (retinal or optic atrophy, cognitive disorder or psychosis, seizures, stroke, cranial neuropathy, and transverse myelitis).

### Transesophageal echocardiography

All 17 participants underwent initial and follow-up two-dimensional (2D) TEE imaging and 10 of them also underwent initial and follow-up three dimensional (3D) TEE imaging with IE-33 Philips systems with digitally acquired images for off-line interpretation and measurements. The detailed methodology of 2D and 3D TEE images acquisition and interpretation has been delineated in previous publications [1–3]. *Libman-Sacks vegetations* by either technique were defined as abnormal localized, protruding, and sessile echodensities >3 mm in diameter with well-defined borders either as part of or adjacent to valve leaflets, annulus, subvalvular apparatus, or endocardial surfaces [1–3]. By 2D and 3D TEE, the maximum diameter and area of vegetations were measured by planimetry. High inter-observer agreement for detection of mitral or aortic valve vegetations by 2D TEE (91%, kappa = 0.80) and 3D TEE (96%, kappa = 0.92) was demonstrated [1, 3]. *Valve regurgitation* was assessed by standard 2D TEE color-Doppler criteria according to guidelines and classified as trivial, mild, moderate, moderate to severe, or severe regurgitation [1, 14]. Similarly, v*alve thickening* was quantitatively assessed using 2D TEE guided M-mode imaging and measured at the base, mid, and distal portions of the anterior mitral leaflet, proximal and distal portions of the posterior mitral leaflet, and body and tip portions of the aortic valve cusps and then averaged [1–3, 15].

### Transcranial doppler

During initial and follow-up studies, both middle cerebral arteries were interrogated for 90 minutes for detection of microembolism defined as audible, high intensity (>13db), and <100ms unidirectional signals within both Doppler blood flow velocity and vessel lumen [16].

### Brain MRI-MRA

Initial and follow-up standard T1-weighted, fluid attenuated inversion recovery (FLAIR), and diffusion-weighted images were obtained. Dynamic susceptibility contrast MRI was also performed to assess brain perfusion [1, 17]. *Focal brain injury* was defined as recent or old cerebral infarcts or ≥5 small focal periventricular or deep white abnormalities. *Hemispheric and whole brain lesion load* in cm^3^ was determined using semi-automated methods [18]. Quantitative assessment of brain perfusion and qualitative and quantitative assessment of brain injury were performed by 3 independent experts, respectively.

### Neurocognitive evaluation

Participants underwent initial and follow-up neurocognitive testing for attention, memory, motor function, processing speed, executive function, and global neurocognitive function [19].

### Anti-inflammatory and Anti-thrombotic therapy in individual SLE patients with Libman-Sacks endocarditis and acute cerebrovascular disease

As shown in **Table 1**, among the 17 patients with Libman-Sacks endocarditis and acute CVD, 14 (82%) had stroke or TIA, 15 (88%) had focal brain injury on MRI, and 10 of 16 (63%) had global neurocognitive dysfunction (one patient with acute stroke could not undergo formal neurocognitive testing). All 17 patients were treated with anti-inflammatory therapy [hydroxychloroquine in 15 (88%), prednisone in 16 (94%), and mycophenolate mofetil, methotrexate, cyclophosphamide, or rituximab in 9 (53%)], 15 (88%) of them with combined therapy. All 17 patients were also treated with antithrombotic therapy (7 with warfarin, 10 with aspirin, 5 with clopidogrel), 5 (29%) of them with combined therapy.

**Table 1:** Anti-inflammatory and Anti-thrombotic therapy in SLE patients with Libman-Sacks endocarditis and acute cerebrovascular disease.

Patient’s Age/Sex	Type of CVD			*Duration of Therapy	Type of Medical Therapy					
	Acute Neurologic Syndrome	Focal Brain Injury	Neurocognitive Dysfunction		HCQ (N = 15)	Prednisone (N = 16) Average mg/day	†Cytotoxics (N = 9)	Warfarin (N = 7)	ASA (N = 10)	Clopidogrel (N = 5)
47/M	Stroke	Yes	Yes	29.9	Yes	10	Yes	Yes	Yes	No
30/F	TIA	Yes	No	3.11	Yes	5	No	No	Yes	No
50/F	TIA	Yes	Yes	18.46	Yes	5	Yes	Yes	Yes	No
41/F	TIA	Yes	No	6.0	Yes	5	No	No	Yes	Yes
57/M	ACS	Yes	Yes	7.08	Yes	10	No	No	Yes	No
30/F	TIA	No	No	2.07	Yes	5	No	No	Yes	No
38/F	ACD	Yes	Yes	9.48	Yes	10	No	Yes	No	No
29/M	TIA	Yes	Yes	7.34	Yes	5	Yes	No	Yes	Yes
27/F	Seizures	No	No	3.84	Yes	15	No	No	Yes	No
38/F	Stroke	Yes	Yes	1.61	Yes	10	Yes	Yes	No	No
24/F	TIA	Yes	Yes	21.1	Yes	10	Yes	Yes	No	No
18/F	Stroke	Yes	Yes	2.20	Yes	5	Yes	Yes	No	No
38/F	Stroke	Yes	Yes	1.02	Yes	20	Yes	No	No	Yes
25/F	Stroke	Yes	No	9.64	Yes	10	Yes	No	No	Yes
51/F	TIA	Yes	No	12.39	No	No	No	No	Yes	No
49/F	TIA	Yes	Yes	2.07	Yes	20	Yes	No	Yes	Yes
31/F	Stroke	Yes	**NS	0.25	No	††Yes	No	Yes	No	No

*Duration in months of medical therapy from initial to follow-up TEE.

**This patient did not undergo formal neurocognitive testing due to her acute stroke syndrome.

†Cytotoxics = included cyclophosphamide, mycophenolate mofetil, methotrexate, or retuximab.

††Intravenous methylprednisolone

Abbreviations: CVD = cerebrovascular disease, HCQ = hydroxycloroquine, ASA = aspirin, M = male, F = female, TIA = transient ischemic attack, ACS = acute confusional state, ACD = acute cognitive dysfunction.

### Statistical analysis

Patients’ baseline descriptive statistics were mean ± SD, or median and interquartile ranges in asymmetrically distributed variables, or frequencies (%). Neurocognitive z-scores were computed using healthy controls as reference. From initial to follow-up studies, the effect of therapy on parameters of inflammation and thrombogenesis, Libman-Sacks endocarditis (valve vegetations, regurgitation, and thickness), cerebrovascular disease (cerebral microembolism, perfusion, and lesion load), and cognitive dysfunction were all assessed by Wilcoxon’s signed rank test as a robust, non-parametric paired comparison. For binary variables, paired comparisons were assessed by 1-sample binomial test. Cerebromicroembolic counts were modeled as Poisson counts. SAS 9.4 was used for all statistical analyses. Two tailed p-values ≤0.05 were considered significant.

## Results

### Effect of medical therapy on parameters of inflammation and thrombogenesis

From initial to follow-up studies, the total, neuro, and non-neuro SLEDAI-2K scores, erythrocyte sedimentation rate, and levels of P-selectin (a cell adhesion molecule indicative of platelet activation and aggregation and endothelial cells activation or injury) decreased (all p≤0.04) while platelet count and levels of plasminogen activator inhibitor (this last change is indicative of increased fibrinolysis) and complement C4 increased (all p≤0.05) (**Table 2**).

**Table 2:** Effect of medical therapy on clinical and laboratory parameters in SLE patients with Libman-Sacks endocarditis and cerebrovascular disease (N = 17).

Characteristic	Enrollment	Follow-up	*P value
Total SLEDAI-2K (units)	18.80 ± 10.35	7.40 ± 8.95	0.0009
Neurologic SLEDAI-2K (units)	11.73 ± 5.12	3.20 ± 5.06	0.002
Non-Neurologic SLEDAI-2K (units)	7.73 ± 6.64	4.20 ± 5.12	0.004
Total SDI (units)	3.80 ± 1.93	3.73 ± 2.02	1.00
Neurologic SDI (units)	1.47 ± 1.25	1.40 ± 1.24	1.00
Non-Neurologic SDI (units)	2.40 ± 1.45	2.40 ± 1.50	1.00
White blood cell count (x10^^3^/mm^3^)	6.31 ± 1.91	6.65 ± 3.21	0.90
Hemoglobin (g/dL)	12.24 ± 2.15	13.07 ± 1.53	0.13
Platelet count (x10^^3^/mm^3^)	196.42 ± 83.56	273.0 ± 95.22	0.03
Creatinine (mg/dL)	0.94 ± 0.40	0.93 ± 0.42	0.70
Albumin (g/dL)	3.65 ± 0.61	3.74 ± 0.42	0.75
dsDNA titer	36.29 ± 58.67	55.71 ± 119.43	0.63
C3 (mg/dL)	76.57 ± 25.66	88.43 ± 32.44	0.44
C4 (mg/dL)	13.07 ± 3.28	18.66 ± 5.70	0.03
CH50 (mg/dL)	48.57 ± 16.66	67.57 ± 23.91	0.16
Fibrinogen (mg/dL)	380.57 ± 60.56	363.86 ± 51.45	0.63
C-reactive protein (mg/dL)	1.07 ± 1.06	0.50 ± 0.22	0.06
Erythrocyte sedimentation rate (mm/hr)	46.86 ± 30.57	23.57 ± 16.52	0.03
IgM anticardiolipin antibody (units)	18.86 ± 11.35	14.86 ± 8.69	0.44
IgG anticardiolipin antibody (units)	49.51 ± 44.85	44.97 ± 44.14	0.63
IgA anticardiolipin antibody (units)	6.60 ± 4.68	6.97 ± 4.79	0.63
P selectin (ng/dL)	51.61 ± 35.07	34.98 ± 11.81	0.03
Plasminogen Activator Inhibitor (U/mL)	8.95 ± 13.14	18.44 ± 17.69	0.003
Tissue Plasminogen Antigen (ng/mL)	11.18 ± 4.28	12.73 ± 7.48	0.53
Platelet-Derived Microparticles (U/uL)	885.20 ± 758.73	609.95 ± 718.99	0.38
Monocyte-Derived Microparticles (U/uL)	432.14 ± 412.14	239.57 ± 410.13	0.12

Cell formats are mean ± SD (n) or frequency/n (%).

*P values by Wilcoxon Signed Rank test.

Abbreviations: DNA = double stranded nuclear antibody, SLE = systemic lupus erythematosus, SLEDAI-2K = SLE disease activity index, SDI = Systemic Lupus International Collaborating Clinics/American College of Rheumatology Damage Index.

### Effect of medical therapy on Libman-Sacks endocarditis

**Fig 2:**
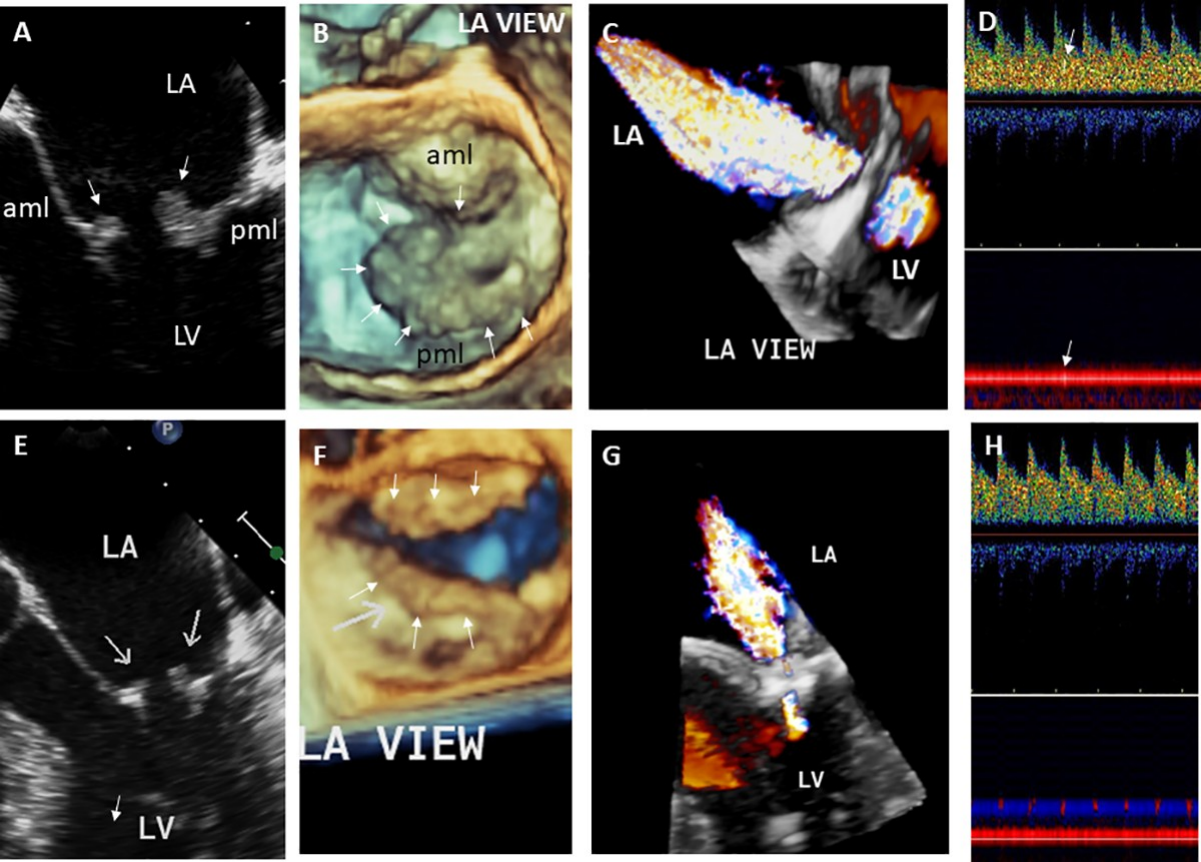
29-year- old male with Libman-Sacks endocarditis complicated with acute stroke and cognitive dysfunction. **A.** This two-dimensional (2-D) TEE view demonstrates large (area of 1.5 cm^2^), sessile, and oval shaped Libman-Sacks vegetations on the distal portions and atrial side of the anterior (aml) and posterior (pml) mitral leaflets (arrows). **B.** This 3-dimensional (3-D) TEE let atrial (LA) view of the mitral valve demonstrates large (area of 3.20 cm^2^) and protruding vegetations on the tip to mid portions and atrial side of the entire anterior and posterior mitral leaflets (arrows). **C**. Associated severe mitral regurgitation is demonstrated by 3D-TEE color Doppler. **D**. Transcranial Doppler of the middle cerebral artery demonstrates a microembolic event within the spectral Doppler (upper arrow) and within the vessel (lower arrow). On MRI, multiple small white matter lesions totaling a lesion load of 4.3 cm^3^ were demonstrated. His neurocognitive dysfunction was graded as severe. After 3 months of aspirin and clopidogrel, hydroxychloroquine, prednisone, and mycophenolate mofetil, mitral valve vegetations significantly decreased in size (arrows) by 2D (**E**) and 3D TEE (**F**) to vegetation areas of 0.22 cm^2^ and 0.79 cm^2^, respectively, and mitral valve regurgitation improved to mild (**G**). Also, cerebromicroembolism resolved (**H**), his brain lesion load decreased to 2.1 cm^3^, and his neurocognitive dysfunction improved to moderate degree. Abbreviations: LA = left atrium, LV = left ventricle, aml = anterior mitral leaflet, pml = posterior mitral leaflet.

**Fig 3:**
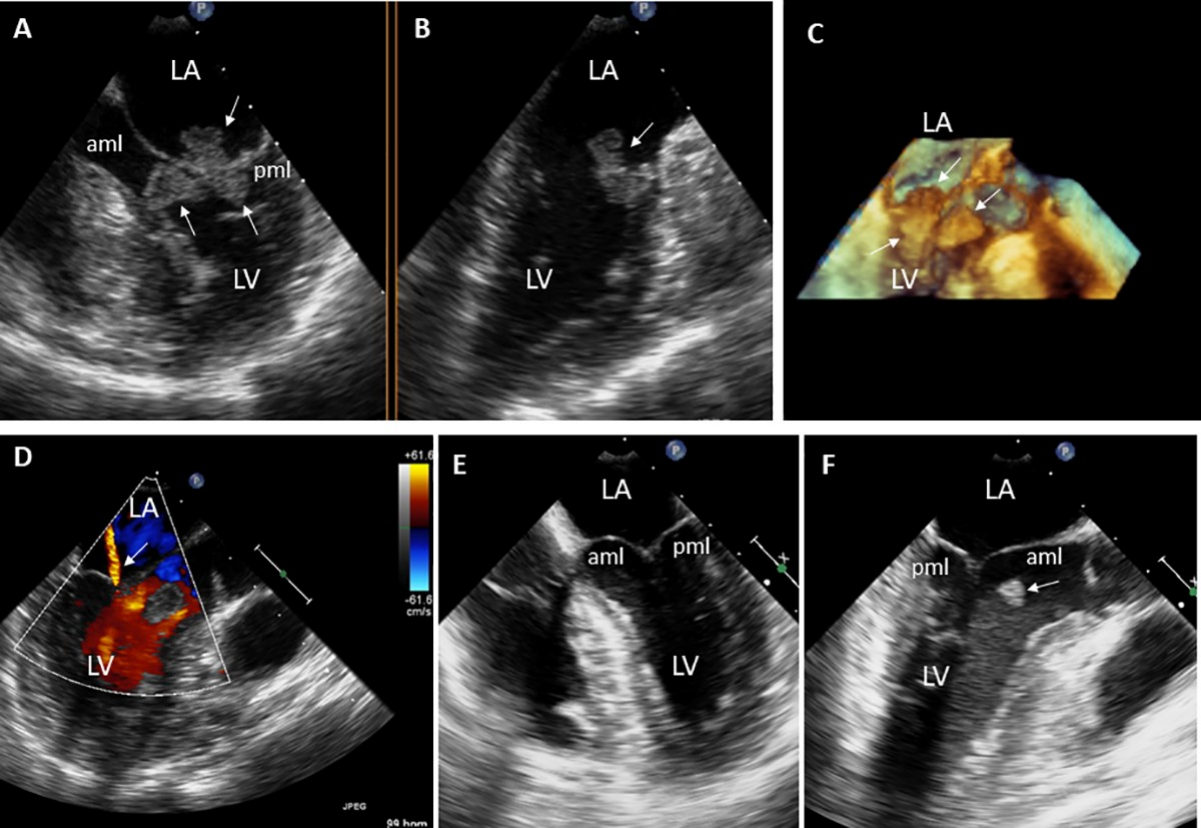
31-year-old female with Libman-Sacks endocarditis complicated with acute stroke. A. This 2-D TEE view demonstrates a large (area of 2.3 cm^2^) and tri-lobed Libman-Sacks vegetation extending from the atrial side of the mitral leaflets into the chorda tendinae (arrows). B. A second large (area of 1.1 cm^2^) vegetation is seen attached to the basal anterior wall of the left ventricle (arrow). C. This 3D-TEE view illustrates further the large and multilobed mitral valve vegetation extending from the atrial side of the mitral valve into the chordae tendinae as well as the anterior wall vegetation (arrows). D. Color Doppler TEE demonstrates only mild mitral regurgitation (arrow). E,F. After 5 days of intravenous heparin and methylprednisolone, follow-up 2D-TEE demonstrates resolution of the mitral valve vegetations (E) and significantly smaller anterior wall vegetation (F, arrow). Abbreviations as in previous Figure.

**Fig 4:**
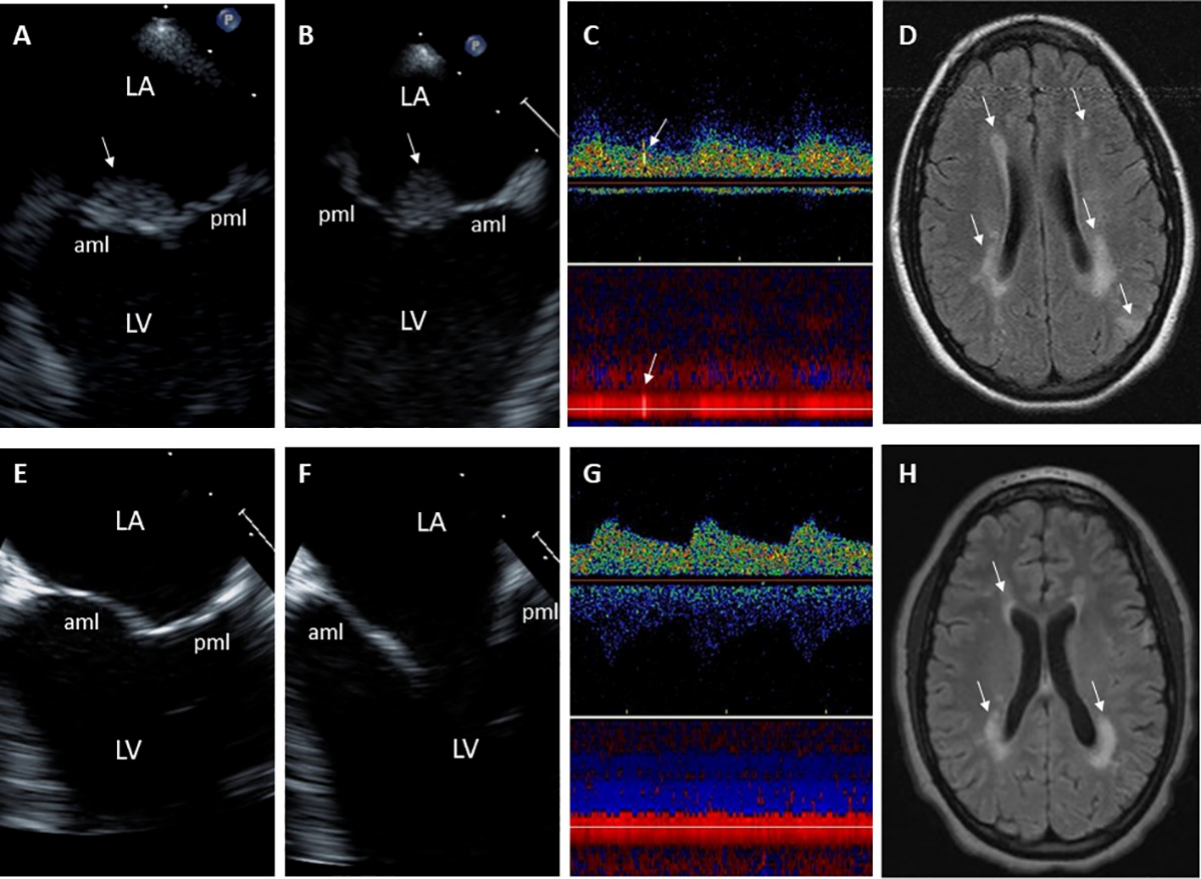
18-year-old female with Libman-Sacks Endocarditis complicated with acute homonymous hemianopsia, confusional state, and cognitive dysfunction. **A,B.** These 2-D TEE orthogonal views (**A,B**) demonstrate a large (area of 1.3 cm^2^), sessile, and oval shaped Libman-Sacks vegetation (arrows) on the atrial side of the anterior mitral leaflet (aml). Associated moderate mitral regurgitation was present. **C**. Transcranial Doppler of the right middle cerebral artery demonstrates 1 of 4 microemboli (arrows). **D**. Diffuse weighted imaging of the brain demonstrates bilateral periventricular cerebral infarcts (arrows) for a total lesion load of 13.71 cm^3^. Her global neurocognitive dysfunction was graded as moderate. Repeat studies after 9 weeks of warfarin, prednisone, and cyclophosphamide showed resolution of the mitral valve vegetation (**E,F**), mitral regurgitation, and cerebromicroembolism (**G**), improvement in brain lesions (**H**) with a decrease of brain lesion load to 7.72 cm^3^, and improvement in her global neurocognitive dysfunction to mild degree. Abbreviations as in previous Figure.

**Fig 5:**
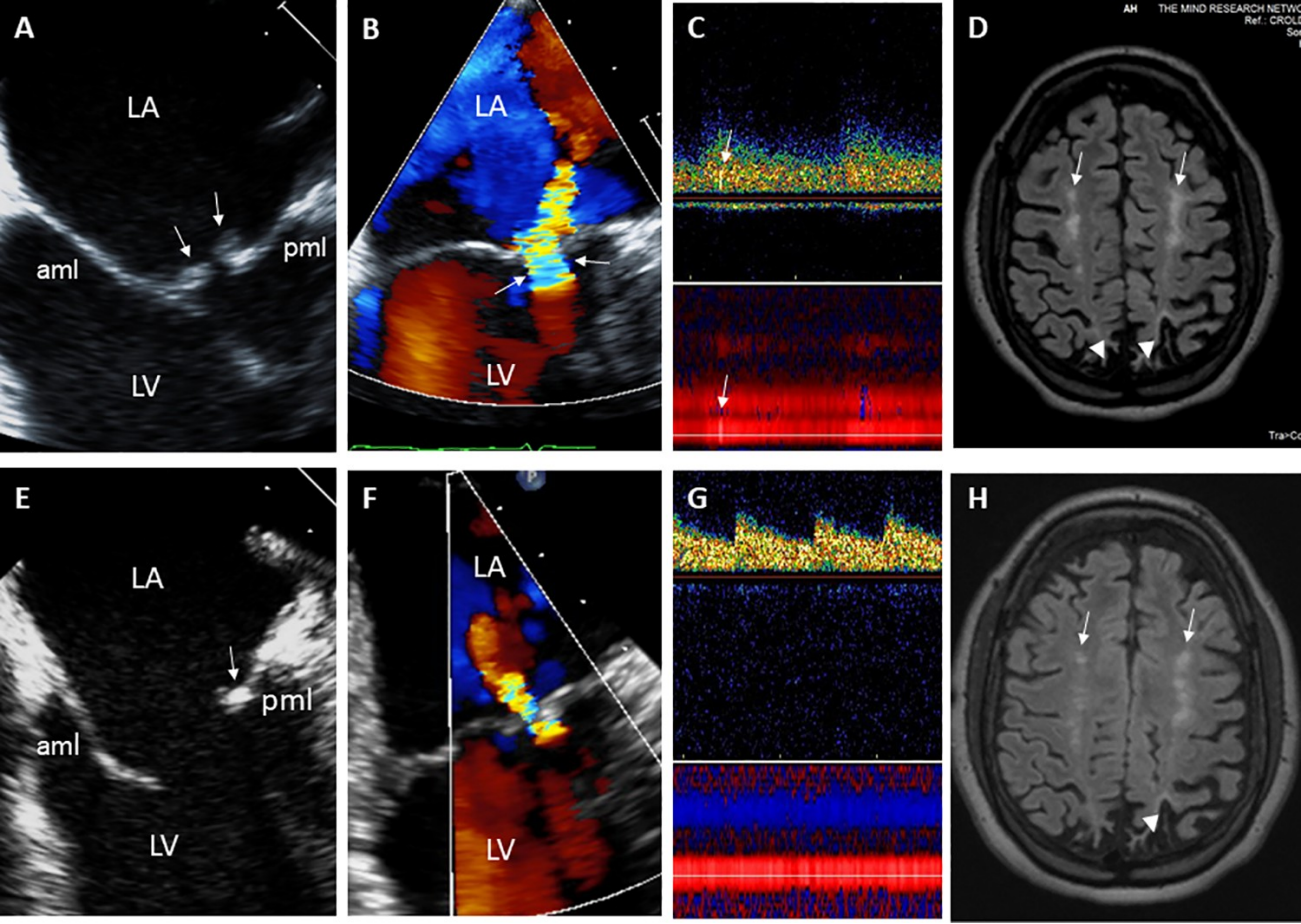
21-year-old female with Libman-Sacks Endocarditis complicated with acute transient ischemic attack and previous stroke. **A,B**. These 2D and color-Doppler TEE views demonstrate a moderate size (area 0.85 cm^2^), sessile, and oval shaped Libman-Sacks vegetations (arrows) on the distal portions and atrial side of the anterior (aml) and posterior (pml) mitral leaflets (**A**) and moderate to severe eccentric mitral regurgitation as demonstrated by a large flow convergence zone (arrows) (**B**). **C**. Transcranial Doppler of the left middle cerebral artery demonstrates 1 of 4 microemboli (arrows). **D.** On MRI, cerebral infarcts (arrowheads) and multiple white matter lesions (arrows) for a lesion load of 7.05 cm^3^ were demonstrated. Her global neurocognitive dysfunction was graded as severe. **E,F.** After 3 months of warfarin, hydroxychloroquine, prednisone, and mycophenolate mofetil, repeat imaging demonstrated resolution of the anterior mitral valve vegetation and reduction in size (arrow) of the posterior mitral leaflet vegetation (**E**), reduction of mitral regurgitation to mild degree (**F**), resolution of cerebromicroembolism (**G**), and decrease in count and size of white matter lesions (arrows in **H**) to a lesion load of 4.2 cm^3^, and improvement in her global neurocognitive dysfunction to mild degree.

**Fig 6:**
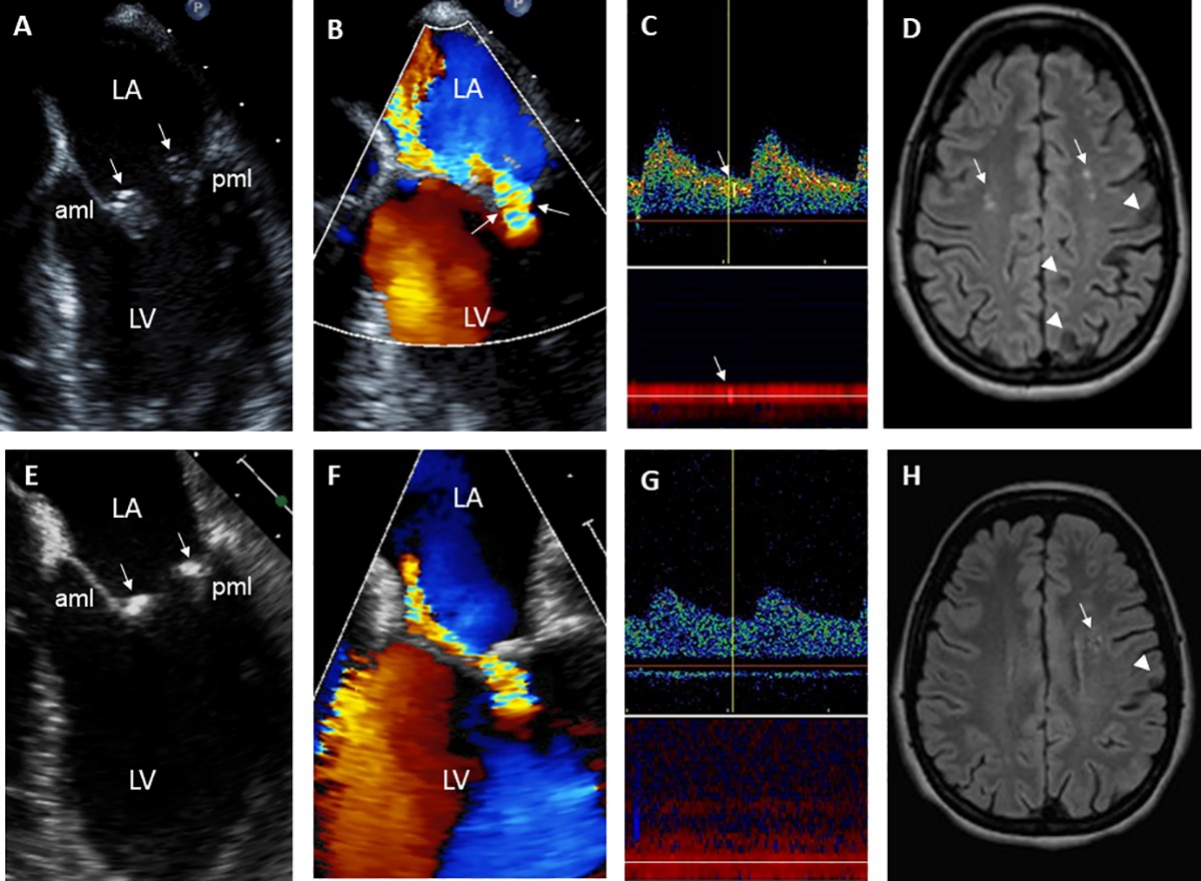
50 year old female with Libman-Sacks endocarditis complicated with an acute transient ischemic attack, past stroke, and seizure disorder. **A,B.** These 2D and color-Doppler TEE views demonstrate moderate size (area of 0.85 cm^2^), sessile, and oval shaped Libman-Sacks vegetations (arrows) on the distal portions and atrial side of the anterior (aml) and posterior (pml) mitral leaflets (**A**) associated with moderate to severe highly eccentric mitral regurgitation (**B**). **C**. Transcranial Doppler of the middle cerebral artery demonstrates 1 of 2 microemboli (arrows). **D.** MRI of the brain demonstrates 3 cerebral infarcts (arrowheads) and multiple small periventricular and deep white matter abnormalities (arrows) for a lesion load of 1.2 cm^3^. Her global neurocognitive dysfunction was graded as moderate. **E,F**. After 5 months of warfarin, aspirin, hydroxychloroquine, and steroids, repeat TEE demonstrated significantly smaller (area 0.13 cm^2^) and homogeneously hyperreflectant indicative of healed mitral valve vegetations (**E**) and improved mitral regurgitation to moderate in degree (**F**). **G**,**H**. She also had no cerebromicroemboli (**G**), her number of brain lesions (**H**) and lesion load decreased to 0.7 cm^3^, and her neurocognitive dysfunction improved to mild degree.

From initial to follow-up TEE studies, valve vegetations decreased in number, diameter, and area (all p≤0.01); the severity of associated valve regurgitation also improved (p = 0.04), and valve thickening decreased, but not significantly (p = 0.56) (**Tables 3 and 4, Figs 2–6**). In 13 (76%) patients, valve vegetations or valve regurgitation resolved or improved in number and size or by ≥1 degree, respectively, as compared to 4 (24%) patients in whom valve vegetations or regurgitation persisted unchanged or increased in size or by ≥1 degree (p = 0.03).

**Table 3:** Effect of medical therapy in patients with Libman-Sacks endocarditis.

Finding	Initial TEE Study (N = 17)	Follow-up TEE Study (N = 17)	*P value
	mean ± SD (range)		
**Vegetations’ Number (n)	2.47 ± 1.33 (1, 5)	1.41 ± 1.28 (0, 4)	0.008
Mitral valve (n)	1.88 ± 1.45 (0, 4)	0.88 ± 0.93 (0, 3)	0.004
Aortic valve (n)	0.59 ± 0.62 (0, 2)	0.53 ± 0.72 (0, 2)	1.0
Vegetations’ Area (cm^2^)	0.61 ± 0.84 (0.04, 3.40)	0.19 ± 0.18 (0, 0.69)	0.01
Mitral valve (cm^2^)	0.55 ± 0.87 (0, 3.40)	0.13 ± 0.18 (0, 0.69)	0.005
Aortic valve (cm^2^)	0.06 ± 0.09 (0, 0.31)	0.06 ± 0.08 (0, 0.20)	0.76
Vegetations’ Diameter (maximum diameter, mm)	7.84 ± 8.43 (2.5, 38.0)	2.93 ± 2.86 (0, 8.10)	0.001
Mitral valve (mm)	6.71 ± 9.06 (0, 38)	2.53 ± 2.75 (0, 8.10)	0.007
Aortic valve (mm)	2.15 ± 2.45 (0, 6.50)	1.39 ± 2.20 (0, 6.10)	0.74
†Valve regurgitation grade (0–5)	1.03 ± 0.59 (0.5, 2.5)	0.69 ± 0.40 (0, 1.5)	0.04
Mitral valve regurgitation grade	1.94 ± 1.18 (1, 5)	1.25 ± 0.68 (0, 2)	0.03
Aortic valve regurgitation grade	0.12 ± 0.34 (0, 1)	0.12 ± 0.34 (0, 1)	1
Valve Thickness (mm)	2.16 ± 0.75 (0.94, 3.87)	2.0 ± 0.52 (1.25, 3.14)	0.56
Mitral valve thickness (mm)	2.56 ± 1.21 (0.53, 5.59)	2.24 ± 0.64 (1.21, 3.21)	0.3
Anterior mitral leaflet thickness (mm)	2.49 ± 1.12 (0.49, 4.43)	2.18 ± 0.69 (1.15, 3.31)	0.33
Posterior mitral leaflet thickness (mm)	2.53 ± 1.68 (0.57, 7.97)	2.35 ± 0.82 (1.22, 4.23)	0.45
Aortic valve thickness (mm)	1.72 ± 0.45 (1.23, 2.94)	1.78 ± 0.54 (1.16, 3.43)	0.52
Right coronary cusp thickness (mm)	1.75 ± 0.41 (1.27, 2.50)	1.78 ± 0.53 (1.12, 3.22)	0.79
Left coronary cusp thickness (mm)	1.79 ± 0.88 (1.13, 4.17)	1.92 ± 0.89 (1.23, 3.97)	0.94
Non-coronary cusp thickening (mm)	1.70 ± 0.63 (1.12, 3.30)	1.78 ± 0.69 (0.93, 3.10)	0.81

Cell format is mean ± SD (range).

*All P values by Wilcoxon Signed Rank test.

**Includes patients with either mitral or aortic valve vegetations present on the initial study.

†Valve regurgitation includes patients with either mitral or aortic regurgitation graded as: 0 = absent, 1 = trivial, 2 = mild, 3 = moderate, 4 = moderate to severe, 5 = severe

TEE = transesophageal echocardiography. Other abbreviations as in previous Tables.

**Table 4:** Effect of medical therapy in patients with Libman-Sacks vegetations assessed by three-dimensional TEE.

Finding	Initial TEE (N = 10)	Follow-up TEE (N = 10)	*P value
	mean ± SD (range)		
**Vegetations’ Number (n)	2.40 ± 1.65 (1, 5)	1.40 ± 1.26 (0, 4)	0.07
Mitral valve (n)	1.90 ± 1.85 (0, 5)	0.50 ± 0.97 (0, 3)	0.02
Aortic valve (n)	1.10 ± 1.52 (0, 5)	1.0 ± 1.05 (0, 3)	1.0
Vegetations’ Area (cm^3^)	0.95 ± 0.99 (0.13, 3.20)	0.21 ± 0.26 (0, 0.79)	0.002
Mitral valve (cm^3^)	1.10 ± 1.18 (0.08, 3.20)	0.17 ± 0.29 (0, 0.79)	0.02
Aortic valve (cm^3^)	0.40 ± 0.29 (0.10, 0.72)	0.17 ± 0.22 (0, 0.49)	0.06
Vegetations’ Diameter (maximum diameter, mm)	10.06 ± 9.88 (4.10, 36.80)	3.37 ± 4.20 (0, 13.30)	0.002
Mitral valve (mm)	11.36 ±11.72 (4.10, 36.80)	3.01 ± 4.91 (0, 13.30)	0.02
Aortic valve (mm)	5.90 ± 2.48 (4.0, 9.90)	2.52 ± 2.84 (0, 6.80)	0.06

Cell format is mean ± SD (range).

*All P values by Wilcoxon Signed Rank test.

**Includes patients with either mitral or aortic valve vegetations present on the initial study.

### Effect of medical therapy on acute cerebrovascular disease

From initial to follow-up studies, cerebromicroembolism, lobar and global gray and white matter cerebral perfusion, ischemic brain lesion load, and neurocognitive dysfunction resolved or improved (all p ≤0.04) (**Table 5, Figs 2 and 4–6**).

**Table 5:** Effect of medical therapy in patients with acute cerebrovascular disease.

Finding	Initial Study (N = 17)	F-Up Study (N = 17)	P value*
**Microembolism**	**Transcranial Doppler, n (%)**		
Right or left middle cerebral artery microembolism	5 patients (29%) with 14 microemboli	0	0.007*
	3 patients with 2 microemboli		
	2 patients with 4 microemboli		
**Brain Perfusion Location**	**Perfusion** (ml/min/100 grams/tissue), (**Mean** ± **SD)**		**Δ%/P value**†
Frontal lobe (gray matter)	26.45 ± 17.58	32.19 ± 14.62	36% / 0.02
Left frontal lobe (gray matter)	26.53 ± 17.55	32.39 ± 14.65	37% / 0.02
Right frontal lobe (gray matter)	26.37 ± 17.61	31.98 ± 14.62	35.6% / 0.02
Frontal lobe (white matter)	12.76 ± 9.14	15.60 ± 6.53	40% / 0.01
Left frontal lobe (white matter)	12.51 ± 8.98	15.36 ± 6.66	39.8% / 0.02
Right frontal lobe (white matter)	13.01 ± 9.30	15.83 ± 6.42	39.2% / 0.01
Parietal lobe (gray matter)	29.25 ± 19.16	35.78 ± 15.20	38% / 0.02
Left parietal lobe (gray matter)	29.13 ± 19.28	35.81 ± 15.31	38.4% / 0.02
Right parietal lobe (gray matter)	29.36 ± 19.04	35.76 ± 15.11	36.9% / 0.02
Parietal lobe (white matter)	13.87 ± 9.02	17.21 ± 6.19	41% / 0.02
Left parietal lobe (white matter)	13.90 ± 8.72	17.47 ± 6.33	41.4% / 0.02
Right parietal lobe (white matter)	13.84 ± 9.35	16.94 ± 6.08	40.8% / 0.02
Temporal lobe (gray matter)	28.23 ± 16.97	33.78 ± 15.21	31% / 0.04
Left temporal lobe (gray matter)	27.72 ± 17.28	33.37 ± 15.55	31.3% / 0.02
Right temporal lobe (gray matter)	28.75 ± 16.70	34.19 ± 14.93	30.3% / 0.049
Temporal lobe (white matter)	14.70 ± 10.31	17.85 ± 7.68	38% / 0.02
Left temporal lobe (white matter)	14.58 ± 9.98	17.75 ± 7.68	36.6% / 0.02
Right temporal lobe (white matter)	14.82 ± 10.63	17.95 ± 7.71	38.8% / 0.02
Occipital lobe (gray matter)	28.46 ± 18.62	33.73 ± 15.39	32% / 0.03
Left occipital lobe (gray matter)	27.88 ± 19.15	33.50 ± 15.94	34.8% / 0.02
Right occipital lobe (gray matter)	29.03 ± 18.11	33.96 ± 14.91	29.0% / 0.03
Occipital lobe (white matter)	16.10 ± 10.56	19.21 ± 7.27	35% / 0.02
Left occipital lobe (white matter)	15.81 ± 10.42	19.09 ± 7.48	35.5% / 0.01
Right occipital lobe (white matter)	16.39 ± 10.74	19.33 ± 7.13	34.0% / 0.02
**Brain Lesion Load Location**	**Lesion Load, (Mean ± SD)**		**P value**†
Whole brain lesion load (cm^3^)	2.70 ± 4.11	1.50 ± 2.34	0.03
Left hemisphere (cm^3^)	1.14 ± 1.87	0.74 ± 1.21	0.1
Right hemisphere (cm^3^)	1.56 ± 2.46	0.76 ± 1.17	0.003
**Cognitive Clinical Domain**	**Neurocognitive z-scores (Mean ± SD)**		**P value**†
Attention	-3.74 ± 4.30	-2.44 ± 3.37	0.003
Memory	-1.73 ± 1.57	-1.11 ± 1.57	0.003
Visual memory	-1.43 ± 1.14	-0.74 ± 1.51	0.01
Verbal memory	-1.99 ± 2.53	-1.44 ± 2.02	0.03
Memory recollection	-0.47 ± 1.56	-0.59 ± 1.17	0.85
Motor function	-6.70 ± 10.73	-2.68 ± 2.67	0.008
Motor Peg	-7.88 ± 10.82	-5.62 ± 7.99	0.04
Motor Tap	-1.43 ± 1.02	-1.36 ± 1.11	0.04
Processing speed	-2.25 ± 2.04	-1.93 ± 2.49	0.06
Global cognitive dysfunction	-3.29 ± 3.11	-2.10 ± 2.38	<0.001

*Poisson regression with repeated measures.

†All P values by Wilcoxon Signed Rank test.

Five patients died from sepsis (2), myocardial infarction (1), posterior leukoencephalopathy (1), and ischemic stroke (1) before they could undergo re-evaluations. These 5 patients as compared to the 17 study patients were significantly older (50.20 ± 7.33 versus 34.37 ± 12.18, p = 0.01) and sicker since they had higher total SLEDAI-2K (20.0 ± 17.48 versus 2.44 ± 3.93, p = 0.04) and a trend toward higher non-neuro-SLEDAI-2K (8.80 ± 8.76 versus 2.59 ± 4.0, p = 0.08) and non-neuro SDI scores (4.20 ± 2.28 versus 2.24 ± 1.60, p = 0.07).

## Discussion

Although expert consensus and clinical guidelines are available for medical therapy of SLE-related cutaneous vasculitis, discoid lupus, interstitial lung disease, alveolitis, arthritis, nephritis, pulmonary hypertension, pericarditis, antiphospholipid syndrome, and neuropsychiatric SLE [7–9, 20], no expert consensus nor guidelines exist for the specific treatment of Libman-Sacks endocarditis and its associated CVD. However, clinicians and surgeons generally advise surgical treatment of Libman-Sacks endocarditis complicated with significant valvular dysfunction or large vegetations and embolization without considering the potential role of anti-inflammatory and anti-thrombotic therapy in resolving or improving this valvulopathy. Therefore, the role of medical therapy for Libman-Sacks endocarditis is a critical issue that has not been adequately addressed.

This observational cross-sectional and longitudinal study of Libman-Sacks endocarditis complicated with acute CVD and treated with conventional anti-inflammatory and anti-thrombotic therapy revealed 3 major findings: 1) clinical parameters of SLE activity and laboratory parameters of inflammation, platelet activity and aggregation, and fibrinolysis improved; 2) Libman-Sacks vegetations and valve regurgitation resolved or significantly improved and valve thickening did not progress; and 3) associated cerebromicroembolism, brain perfusion, brain lesion load, and neurocognitive dysfunction also resolved or improved. To our knowledge, this is the first study to demonstrate a beneficial effect of anti-inflammatory and anti-thrombotic therapy for Libman-Sacks endocarditis and its associated CVD and suggests that medical therapy might obviate the need for high risk valve surgery in SLE patients.

The rationale and benefit of anti-inflammatory and anti-thrombotic therapy for Libman-Sacks endocarditis is supported not only by its well-defined autoimmune inflammatory and thrombotic pathogenesis on light and immunofluorescent microscopy, but also by the transition on immune-histopathology from active to mixed or healed Libman-Sacks vegetations with consequent decreased risk of embolism [1–3, 10–12]. Active vegetations are those with central myxoid degeneration, fibrinoid necrosis, fibrous connective tissue, and hemorrhages surrounded by fibrinous exudate, proliferating capillaries and fibroblasts, polymorphonuclear cell inflammation and peripherally by proteinaceous material, fibrin deposition, and platelet thrombi. In these vegetations, deposition of immunoglobulins IgG, IgM, and IgA, anticardiolipin antibodies, and C1q, C3, and C4 complement within the walls of vessels in the inner zone of neovascularization are also present. Mixed vegetations are those with intermixed areas of activity and healing with decreased platelet thrombi with variable degrees of hyalinization and endothelialization and decreased degree of immunoglobulins and complement deposition. Healed vegetations are those with fibroblastic proliferation, central fibrosis, neovascularization, minimal to no inflammation, and absent or organized or hyalinized thrombus with partial or full endothelialization, and absent immune-complex deposition.

The beneficial effect of anticoagulation or antiplatelet therapy frequently combined with hydroxychloroquine therapy in SLE patients with antiphospholipid (aPL) syndrome frequently (39% to 77% of cases) manifested with arterial thrombosis (predominantly stroke or TIA) further support our study findings. Randomized controlled trials and retrospective studies in aPL syndrome including 38% to 59% of cases with SLE have demonstrated a significant reduction in the rate of recurrent arterial thrombotic events in patients treated with high or moderate intensity warfarin therapy or with single or dual antiplatelet therapy [21–26]. Although valvular heart disease in these studies was not studied or reported, high prevalence rates of Libman-Sacks endocarditis have been reported using TEE in SLE patients with aPL antibodies [12, 27]. Hydroxychloroquine is now a standard therapy in SLE patients due to its anti-inflammatory, anti-thrombotic, and immunomodulatory effects [28–30]. In a prospective study of 232 SLE patients treated with hydroxychloroquine, chloroquine, or both for a median of 52 months, a significant protective effect against thrombosis was demonstrated (HR 0.28, 95% CI 0.08–0.90) [31]. Finally, few isolated case reports have shown that immunosuppressive therapy combined with anti-thrombotic and hydroxychloroquine therapy resolve or improve Libman-Sacks endocarditis and its associated CVD [32–34].

The surgical treatment of Libman-Sacks endocarditis is predicated on the assumption that this valvular heart disease may be permanent, progressive, or remain as a high-risk substrate for thromboembolism and valve dysfunction. However, the active underlying systemic and endocardial inflammatory and thrombogenic processes present in Libman-Sacks endocarditis are known to predispose patients to post-operative complications such as: 1) embolism of valve vegetations [35]; 2) prosthesis dehiscence [36]; 3) prosthetic valve stenosis and/or regurgitation due to thrombosis, premature degeneration, or pannus formation [37]; 4) infective endocarditis [38]; 5) autoimmune-mediated bioprosthetic valvulitis, perforation, or accelerated degeneration requiring even higher risk re-do valve surgery [36, 37, 39]; and 6) life-long bleeding and teratogenic complications of needed warfarin therapy [40]. As evidence of the high risk of valve surgery in SLE patients, Bouma W, et al [5] reported a review of 41 young SLE patients (68% <50 years old) with Libman-Sacks endocarditis who underwent mitral valve replacement with a mechanical or bioprosthetic valve in 30 (73%) or valve repair in 11 (27%) patients. In 9 (22%) patients, no post-operative data was available. Of the 32 subjects with post-operative data, 4 (13%) died and 8 (25%) others had post-operative stroke or peripheral/visceral embolism, myocardial infarction, valve thrombosis, or development of recurrent valve regurgitation, valve stenosis, or arteriovenous fistula. Also, Foroughi M, et al [6], reported a review of another 84 young SLE patients (80% <50 years old) who underwent valve replacement in 72 (86%) and valve repair in 12 (14%) for Libman-Sacks endocarditis complicated with significant valve dysfunction. No post-operative follow-up data was available in 36 (43%) patients, also suggesting a poor outcome. During a post-operative follow-up period of 2 months to 10 years in the remaining 48 patients, 12 (25%) died and 13 (27%) others had post-operative ischemic and hemorrhagic stroke, transient ischemic attack, myocardial infarction, ventricular arrhythmias, heart failure, sepsis, renal failure, or re-do valve replacement. These 2 important reviews demonstrate 3–5 times higher surgical morbidity (25% - 30%) and mortality (13% - 25%) in SLE patients as compared to those without SLE. These morbidity and mortality rates may be underestimated due to lack of follow-up data in 22% - 43% of patients. Therefore, since Libman-Sacks endocarditis is potentially reversible, an initial aggressive trial of combined anti-inflammatory and anti-thrombotic therapy should be considered before committing these patients to high risk and irreversible valve surgery.

### Study limitations

Although a small population was studied, to the best of our knowledge, this is the first integrated clinical, laboratory, and multimodality imaging, cross-sectional and longitudinal study to demonstrate a beneficial effect of medical therapy for Libman-Sacks endocarditis and its associated CVD. Although medical therapy was not controlled nor standardized, the anti-inflammatory and antithrombotic therapy used in the study conforms with standard clinical practice and recommendations for the treatment of SLE and CVD by the American College of Rheumatology and European League Against Rheumatism [7–9, 20]. Also, a placebo-controlled group may not be ethically feasible. Since only 4 (22%) patients had moderate to severe valve regurgitation, the effect of medical therapy on advanced valve dysfunction may be underrepresented. However, the prevalence rates of significant valve dysfunction described in this study are similar to those reported in previous series and are generally lower than those of embolic CVD in Libman-Sacks endocarditis [1–3, 15].

## Conclusions

The findings of this study suggest that Libman-Sacks endocarditis and associated acute CVD often resolve or significantly improve with conventional anti-inflammatory and antithrombotic therapy. These results argue for a multidisciplinary approach and an initial trial of medical therapy for Libman-Sacks endocarditis with close clinical follow-up and reimaging before considering high-risk valve surgery. However, a larger randomized cross-sectional and longitudinal study of antiplatelet versus anticoagulant therapy combined with standard anti-inflammatory therapy is needed to confirm these findings.

## Supporting information

S1 Dataset (SAS7BDAT)
